# Hemodynamic variability and cerebrovascular control after transient cerebral ischemia

**DOI:** 10.14814/phy2.12602

**Published:** 2015-11-04

**Authors:** Philip D Allan, James Faulkner, Terrence O’Donnell, Jeremy Lanford, Lai-kin Wong, Saqib Saleem, Brandon Woolley, Danielle Lambrick, Lee Stoner, Yu-Chieh Tzeng

**Affiliations:** 1 Centre for Translational Physiology, University of OtagoWellington, New Zealand; 2 Department of Surgery and Anaesthesia, University of OtagoWellington, New Zealand; 3 Department of Sport and Exercise, University of WinchesterWinchester, UK; 4 Department of Neurology, Wellington HospitalWellington, New Zealand; 5 School of Engineering and Computer Science, Victoria University of WellingtonWellington, New Zealand; 6 School of Sport and Exercise, Massey UniversityWellington, New Zealand; 7 Faculty of Health Science, University of SouthamptonSouthampton, UK

**Keywords:** Blood pressure, cerebral blood flow, cerebral hemodynamics, transient ischemic attack

## Abstract

We investigated if hemodynamic variability, cerebral blood flow (CBF) regulation, and their interrelationships differ between patients with transient ischemic attack (TIA) and controls. We recorded blood pressure (BP) and bilateral middle cerebral artery flow velocity (MCAv) in a cohort of TIA patients (*n* = 17), and age-matched controls (*n* = 15). Spontaneous fluctuations in BP and MCAv were characterized by spectral power analysis, and CBF regulation was assessed by wavelet phase synchronization analysis in the very low- (0.02–0.07 Hz), low- (0.07–0.20 Hz), and high-frequency (0.20–0.40 Hz) ranges. Furthermore, cerebrovascular CO_2_ reactivity was assessed as a second metric of CBF regulation by inducing hypercapnia with 8% CO_2_ inhalation followed by hyperventilation driven hypocapnia. We found that TIA was associated with higher BP power (group effect, *P *<* *0.05), but not MCAv power (*P *=* *0.11). CBF regulation (assessed by wavelet phase synchronization and CO_2_ reactivity) was intact in patients (all *P *≥* *0.075) across both hemispheres (all *P *≥* *0.51). Pooled data (controls and affected hemisphere of patients) showed that BP and MCAv power were positively correlated at all frequency ranges (*R*^2^ = 0.20–0.80, all *P *<* *0.01). Furthermore, LF phase synchronization index was a significant determinant of MCAv power (*P *<* *0.05), while VLF and HF phase synchronization index, and TIA were not (all *P *≥* *0.50). These results indicate that CBF stability and control is maintained in TIA patients, but BPV is markedly elevated. BPV attenuation may be an important therapeutic strategy for enhancing secondary stroke prevention in patients who suffer a TIA.

## Introduction

Transient ischemic attack (TIA) is a common medical emergency that frequently heralds a subsequent stroke (Coull et al. 2004). United States estimates of TIA prevalence are as high as 4.1% (women, 75–79 years) (Price et al. 1993), with an incidence of 1.1 per 1000 population (Edlow et al. 2006). Furthermore, TIA increases stroke risk to 8% at 1 week and 11.5% at 1 month following an event (Coull et al. 2004). Therefore, secondary prevention of acute stroke is an important strategy against the rising human and economic cost of stroke (Norrving and Kissela 2013).

The active management of hypertension is an important secondary prevention strategy, but it is possible that other markers of cerebral hemodynamic stability such as blood pressure variability (BPV), cerebral autoregulation (CA), and cerebrovascular CO_2_ reactivity may also be influential. Like hypertension, aberrant increases in BPV (Stead et al. 2006; Ko et al. 2010; Endo et al. 2013) and impairment of CA (Czosnyka et al. 1996; Budohoski et al. 2015) or CO_2_ reactivity (Grosset et al. 1993) are associated with secondary complications and poor clinical outcomes. But the relevance of these changes in TIA remains unclear because most studies on the neurological consequences of hemodynamic instability have centered on acute stroke of moderate or greater severity (e.g., Stead et al. 2006; Endo et al. 2013). Unlike acute stroke, TIAs are transient events whereby the focus of clinical management is around secondary prevention. To the best of our knowledge, there are no detailed hemodynamic studies of TIA patients who are on established secondary prevention therapies.

Therefore, the goal of this study was to establish whether there are differences in hemodynamic status and cerebrovascular control in TIA patients compared to controls. First, we sought to compare the magnitude of BPV and cerebral blood flow variability (CFV) to test the hypothesis that TIA patients have increased hemodynamic variability despite the use of current secondary prevention therapies. Second, we examined mechanisms that are key to maintaining cerebrovascular homeostasis, such as CA and CO_2_ reactivity, to test the hypothesis that TIA would be associated with cerebrovascular impairment. Finally, we sought to identify the key determinants of CFV. To achieve these objectives, we employed multivariate cerebrovascular assessment techniques that account for potent confounders such as the partial pressure of arterial PCO_2_ (Latka et al. 2005). To exclude the effects of cerebrovascular reactivity from the ventilatory response to PCO_2_ (Ainslie and Duffin 2009), we employed a dynamic, rather than steady state, CO_2_ reactivity procedure (Peebles et al. 2012).

## Methods

### Participants

Seventeen male patients (mean age 66.6 ± 11.3 [SD] years) with a clinical diagnosis of TIA (focal neurological symptoms lasting <24 h) were recruited from Wellington Hospital, New Zealand, and studied at between 1 and 2 weeks following hospital presentation. This was done to ensure that patients had been on optimal therapy for at least 1 week. Patients receiving supplemental oxygen, with uncontrolled angina, unstable cardiac condition, significant cognitive impairment, or other major medical condition were excluded. At the time of hospital presentation, stroke risk was assessed using the ABCD^2^ score. Additionally, 15 age-matched male volunteers (mean age 68.9 ± 4.9 years), without a history of TIA, stroke, or other major neurological condition were recruited as controls. All participants provided written informed consent prior to study participation. Ethical approval was given by the New Zealand Central Regional Ethics Committee.

### Measurements

All measurements were conducted in a thermoneutral laboratory (21–22°C), with the participant in the supine position. Measurements comprised: right and left middle cerebral artery flow velocities (MCAv; 2-MHz pulsed Doppler ultrasound, ST3 Digital Transcranial Doppler System, Spencer Technologies, Seattle), blood pressure (BP; Finometer MIDI, MLE1054-V, Finapres Medical Systems B.V., Amsterdam, Netherlands), nasal partial pressure of end-tidal CO_2_ (P_ET_CO_2_), and O_2_ (Gas analyzer ML206, ADInstruments, Colorado Springs) and 3-lead electrocardiogram. All data were acquired continuously at 1 kHz (PowerLab/16SP ML795, ADInstruments) and subsequently analyzed using custom software (LabVIEW 2013, National Instruments Corporation, Austin and Matlab Version R2014a, MathWorks, Natick). Data were excluded from analyses if there were major signal artifacts or the participant was unable to complete the test procedure.

### Protocol

An initial 5-min baseline recording was made for all participants resting in the supine position. Participants then underwent dynamic CO_2_ reactivity testing as previously described. (Peebles et al. 2012). Briefly, participants first inhaled 8% CO_2_ gas followed directly by hyperventilation to, respectively, raise then lower P_ET_CO_2_ by approximately 5 mmHg. In total, this procedure lasted ∼3.5 min. Brachial systolic and diastolic blood pressure (SBP and DBP, respectively) measurements were made using an automated blood pressure monitor (Pulsecor, Uscom, Sydney, Australia).

### Cerebral hemodynamic assessment

BPV and CFV were characterized as the spectral powers of BP and MCAv, respectively. Raw 1 kHz waveforms were decimated to 1 Hz and passed through a Hanning window before undergoing fast Fourier transform analysis based on the Welch algorithm (50% overlap) in the very low- (VLF, 0.02–0.07 Hz), low- (LF, 0.07–0.20 Hz), and high-frequency (HF, 0.20–0.40 Hz) ranges. These bands are commonly defined, and are based on the high-pass filter characteristics of CA (Zhang et al. 1998).

We performed multivariate wavelet phase synchronization analysis to quantify the extent to which BP and MCAv fluctuations were phase synchronized after correcting for CO_2_-mediated phase distortions (Latka et al. 2005; Peng et al. 2010). Phase synchronization index (PSI) values lie between 0 and 1, where 0 represents a uniform distribution of phase difference and 1 is complete phase synchronization termed “phase lock”. Higher synchronization between BP and MCAv reflects a pressure-passive circulation and is indicative of worse CBF regulation. To facilitate comparison to an established method for CA quantification, we also analyzed data using linear transfer function analysis as previously described (Zhang et al. 1998). Transfer function analysis yields three interpretable parameters reflecting the linearity (coherence), magnitude (gain), and timing (phase) relationships between BP and MCAv as a function of frequency. To account for interindividual variations in the middle cerebral artery diameter, MCAv spectral power and gain were expressed in normalized units, defined as the signal divided by its mean. MCAv was also normalized in the same manner for CO_2_ reactivity analysis. A common interpretation of transfer function metrics is that higher values of coherence and gain and lower values of phase are indicative of impaired CA (Zhang et al. 1998). See appendix for further details.

CO_2_ reactivity was quantified as the linear relationship between breath-to-breath P_ET_CO_2_ and average normalized MCAv within successive breaths, after accounting for BP as a known covariate using linear mixed-effects models as previously described (Peebles et al. 2012). First, to ensure that reactivity estimates accounted for inherent delays in the breathing circuit, the P_ET_CO_2_ trace was left shifted relative to both the BP and MCAv signal. Next, to account for the physiological latency of the CO_2_ reactivity response, the time interval corresponding to the maximum positive cross correlation between P_ET_CO_2_ and MCAv was identified, and time shifted to incorporate the delay. Finally, CO_2_ reactivity was assessed using linear mixed-effects models by entering MCAv as the dependent variable, and P_ET_CO_2_ and BP as independent variables. Analyses were conducted separately for the hypercapnic and hypocapnic regions of the P_ET_CO_2_ trace to account for possible differences in vasodilator versus vasoconstrictor responses (Peebles et al. 2012). In contrast to least square regression, mixed-effects models explicitly account for the fact that repeated P_ET_CO_2_ and MCAv measurements made within subjects are correlated in nature and therefore violate the case independence assumption required for least squares regression (Lazic 2010). Moreover, linear mixed-effects models analyze data in a single step, rather than reducing an individual’s data to a summary measure, before secondary analysis. Thus, the approach increases the precision of the final estimate by accounting for the standard error of individual slope estimates (Lazic 2010).

### Statistical analysis

Linear mixed-effects models were used to determine whether hemodynamic, ventilatory, and cerebrovascular parameters differed between patients and controls. For all comparisons, data from the right and left MCAv were averaged for control subjects, but considered separately in patients to identify potential differences between the affected and unaffected hemispheres. Pearson’s *χ*^2^ test was used to compare the categorical demographic data between patients and controls. BP and MCAv spectral powers were log transformed to achieve normal distributions. To investigate the effect of TIA on hemodynamic variability, we tested for group (patient vs. control), frequency (VLF, LF, and HF), and group × frequency interaction effects. To establish if CA was altered by TIA, group (patients vs. controls) and hemisphere (affected vs. unaffected) main effects were analyzed. We also tested for a group × P_ET_CO_2_ interaction effect to establish differences in CO_2_ reactivity between patients and controls, and a hemisphere × P_ET_CO_2_ interaction to establish differences in CO_2_ reactivity between the affected and unaffected cerebral hemispheres. A priori comparisons for baseline versus hypercapnia and hypocapnia P_ET_CO_2_ and MCAv values were conducted using Student’s paired *t*-tests. Relationships between BP power, group status (patient or control), CA integrity, and MCAv power were analyzed using multiple linear regression. All statistical analyses were performed using SPSS (IBM SPSS Statistics 22, Armonk). Unless otherwise stated, all data are expressed as mean ± SD, with alpha defined as *P *≤* *0.05.

## Results

### Participant characteristics

Demographic and baseline recording physiological characteristics are summarized in Table1. Seventeen male patients were studied at a mean of 9.2 ± 3.5 days from hospital presentation. ABCD^2^ score was assessed in 13 TIA patients, mean 3.8 ± 1.5. Body mass index, SBP, and DBP were higher in patients compared to controls (group effect, all *P *<* *0.05), while MCAv in the affected hemisphere, heart rate, P_ET_CO_2_, and cerebrovascular conductance index (CVCi) were similar between groups (group effect, all *P *≥* *0.080). MCAv (52.5 ± 13.2 vs. 49.8 ± 13.7 cm sec^−1^, hemisphere effect *P *=* *0.58), and CVCi (0.63 ± 0.21 vs. 0.60 ± 0.24 cm sec^−1^, hemisphere effect *P *=* *0.72) were also similar between the affected and unaffected hemispheres.

**Table 1:** Demographic and baseline recording physiological characteristics

	Study group		*P* value
	Patients (*n* = 17, male)	Controls (*n* = 15, male)	Group
Age, years	66.6 ± 11.3	68.9 ± 4.9	0.47
BMI, kg m^−2^	30.8 ± 5.8	26.8 ± 2.2	0.017*
SBP, mmHg	141.8 ± 22.6	126.5 ± 15.4	0.035*
DBP, mmHg	81.3 ± 10.5	72.7 ± 10.1	0.026*
MCAv, cm sec^−1^	*Af* 52.5 ± 13.2† *Un* 49.8 ± 13.7†	44.8 ± 9.8	0.080 (H 0.58)
Heart rate, beats min^−1^	62.3 ± 15.2	61.6 ± 7.5	0.88
P_ET_CO_2_, mmHg	34.9 ± 2.8	34.7 ± 4.1	0.86
CVCi, cm sec^−1^ mmHg^−1^	*Af* 0.63 ± 0.21† *Un* 0.60 ± 0.24†	0.57 ± 0.10	0.30 (H 0.72)
Hypertension	11 (64.7)	4 (26.7)	0.031*
Hyperlipidaemia	10 (58.8)	3 (20)	0.026*
Diabetes	4 (23.5)	2 (13.3)	0.46
Antihypertensive treatment	17 (100)	4 (26.7)	<0.0001*
Lipid lowering treatment	16 (94.1)	4 (26.7)	<0.0001*
Antiplatelet treatment	17 (100)	2 (13.3)	<0.0001*

Values are mean ± SD or number of participants (%). BMI, body mass index; SBP, systolic blood pressure; DBP, diastolic blood pressure; MCAv, middle cerebral artery flow velocity; Af, affected hemisphere; Un, unaffected hemisphere; H, hemisphere; P_ET_CO_2_, partial pressure of end-tidal CO_2_; CVCi, cerebrovascular conductance index.

* *P *<* *0.05.

† *n* = 15 affected hemisphere and 16 unaffected hemisphere of patients for MCAv and CVCi comparisons.

Baseline blood pressure data were obtained in all participants. At baseline, bilateral transcranial Doppler failure occurred in one patient and unilateral failure (affected hemisphere) in one patient. Both of these patients did not undergo CO_2_ reactivity testing due to transcranial Doppler failure and a poor finger plethysmography signal, respectively. Additionally, during CO_2_ reactivity testing, unilateral (unaffected hemisphere) transcranial Doppler failure occurred in one patient during hypocania. Further, one patient was unable to complete the hypercapnic portion of CO_2_ reactivity testing. This is summarized in the respective tables and figures.

### Hemodynamic variability

**Figure 1:**
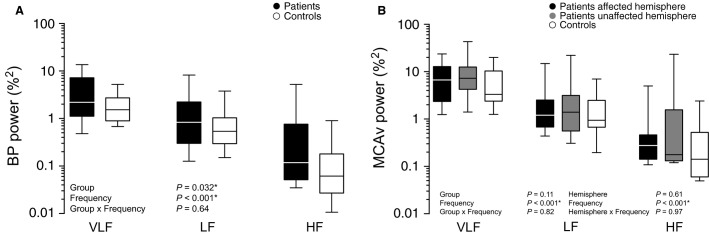
Comparisons for blood pressure and middle cerebral artery flow velocity spectral powers. (A) Patient versus control comparison for BP power. (B) Patient (affected and unaffected hemispheres) versus control comparisons for MCAv power. Spectral powers were log-transformed for statistical analyses, however, for ease of interpretation, they are shown here in raw units on log axes. *P <* *0.05 for group and hemisphere main effect. BP, blood pressure; MCAv, middle cerebral artery flow velocity; VLF, very low-frequency; LF, low-frequency; HF, high-frequency.

Figure1 summarizes the relative magnitudes of hemodynamic variability in the VLF, LF, and HF frequency ranges. A significant group effect was seen for BPV, indicating that patients had greater BPV than controls (*P *<* *0.05) across each of the three frequency ranges (interaction effect, *P *=* *0.64) (97% VLF, 88% LF, and 248% HF higher in patients compared to controls). However, CFV was not increased in patients (group effect, *P *=* *0.11), nor did CFV differ between hemispheres (hemisphere effect, *P *=* *0.61). No group × frequency (*P *=* *0.82) or hemisphere × frequency (*P *=* *0.97) interaction was observed, indicating that the relationship between CFV and frequency was comparable between groups and hemispheres (for patients).

### Cerebral blood flow regulation

**Figure 2:**
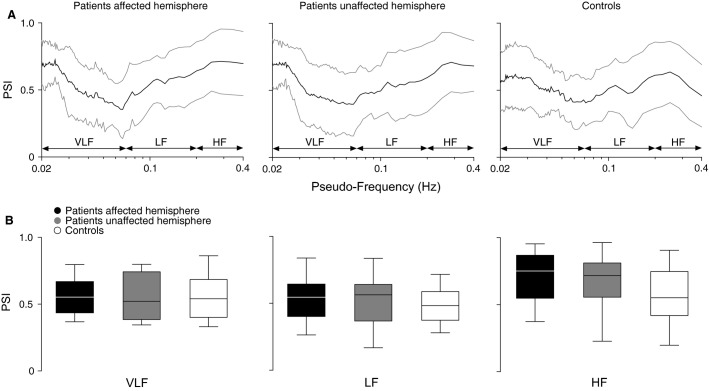
Wavelet phase synchronization between BP and MCAv, with P_ET_CO_2_ as an additional input. (A) PSI against corresponding pseudo-frequencies (representing the result of wavelet scale to frequency conversion). (B) Patient (affected and unaffected hemispheres) versus control comparisons for mean PSI. BP, blood pressure; MCAv, middle cerebral artery flow velocity; P_ET_CO_2_, partial pressure of end-tidal CO_2_; PSI, phase synchronization index; VLF, very low-frequency; LF, low-frequency; HF, high-frequency.

Figure2 shows wavelet phase synchronization between BP and MCAv after correcting for the effects of P_ET_CO_2_ as an additional input. Analyses indicate that PSI did not differ significantly between patients and controls across all frequency bands (group effect, all *P *≥* *0.075), nor between hemispheres (hemisphere effect, all *P *≥* *0.75).

Transfer function results are presented in Table2. There was a significant group effect for HF phase (*P *<* *0.05), but not VLF or LF phase, indicating that only HF phase differed between patients and controls. We did not find a significant group effect for coherence and normalized gain at all frequency ranges (all *P *≥* *0.57). No significant hemisphere effect was found for any transfer function metric at all frequency bands (all *P *≥* *0.59), indicating similar transfer function values for both the affected and unaffected hemispheres of patients.

**Table 2:** Summary of spontaneous baseline transfer function analysis variables

Variable	Study group		*P* value	
	Patients (*n* = 15 [*Af*] and 16 [*Un*])	Controls (*n* = 15)	Group	Hemisphere
VLF coherence	*Af* 0.52 ± 0.11 *Un* 0.53 ± 0.12	0.51 ± 0.17	0.91	0.73
LF coherence	*Af* 0.66 ± 0.21 *Un* 0.71 ± 0.20	0.67 ± 0.15	0.95	0.59
HF coherence	*Af* 0.43 ± 0.19 *Un* 0.43 ± 0.19	0.47 ± 0.15	0.57	0.99
VLF phase, radians	*Af* 0.69 ± 0.34 *Un* 0.75 ± 0.47	0.51 ± 0.47	0.26	0.69
LF phase, radians	*Af* 0.47 ± 0.27 *Un* 0.48 ± 0.22	0.52 ± 0.13	0.86	0.98
HF phase, radians	*Af* 0.0011 ± 0.32 *Un* 0.045 ± 0.32	−0.19 ± 0.13	0.026*	0.75
VLF n-gain, % mmHg^−1^	*Af* 0.97 ± 0.46 *Un* 1.01 ± 0.41	0.98 ± 0.38	0.95	0.75
LF n-gain, % mmHg^−1^	*Af* 1.10 ± 0.37 *Un* 1.10 ± 0.37	1.10 ± 0.27	0.89	0.88
HF n-gain, % mmHg^−1^	*Af* 1.24 ± 0.40 *Un* 1.22 ± 0.39	1.21 ± 0.26	0.85	0.95

Values are mean ± SD. VLF, very low-frequency; LF, low-frequency; HF, high-frequency; Af, affected hemisphere; Un, unaffected hemisphere; n-gain, normalised gain.

* *P *<* *0.05 for main effect for group and hemisphere.

Table3 presents P_ET_CO_2_ and MCAv values obtained during CO_2_ reactivity testing. The hypercapnia and hypocapnia procedures caused increases and decreases in P_ET_CO_2_, with consistent changes in MCAv in patients (both hemispheres) and controls. CO_2_ reactivity analyses are summarized in Table4. Group × P_ET_CO_2_ interaction effects were not seen for hypercapnic or hypocapnic CO_2_ reactivity (all *P *≥* *0.53), indicating that CO_2_ reactivity did not differ between patients and controls. CO_2_ reactivity was similar between patient hemispheres across all stages of CO_2_ reactivity (interaction effect, all *P *≥* *0.51).

**Table 3:** Partial pressures of end-tidal CO_2_ and middle cerebral artery flow velocities during CO_2_ reactivity

	Study group					
	Patients (*n* = 15 [*Af*] and 14 [*Un*])*			Controls (*n* = 14)		
	Baseline	Hypercapnia	Hypocapnia	Baseline	Hypercapnia	Hypocapnia
P_ET_CO_2_, mmHg	31.7 ± 4.4	37.0 ± 5.1†	26.2 ± 4.0†	30.1 ± 5.1	35.2 ± 5.4†	25.1 ± 4.6†
MCAv, cm sec^−1^	*Af* 42.8 ± 11.9 *Un* 40.7 ± 13.2	*Af* 54.9 ± 17.1† *Un* 51.9 ± 16.1†	*Af* 34.7 ± 6.4† *Un* 33.1 ± 7.0†	38.1 ± 9.2	46.1 ± 11.5†	32.0 ± 7.3†

Values are mean ± SD. Baseline refers to the 30 sec immediately preceding the hypercapnia procedure. Hypercapnia and hypocapnia refer to the maximum and minimum P_ET_CO_2_ respectivley, during CO_2_ reactivity testing. MCAv data is presented in raw units for ease of interpretation, but was normalised for CO_2_ reactivity analysis. Af, affected hemisphere; Un, unaffected hemisphere; P_ET_CO_2_, partial pressure of end-tidal CO_2_; MCAv, middle cerebral artery flow velocity.

* For hypocapnia response. For hypercapnia, *n* = 14 affected hemisphere and 13 unaffected hemisphere of patients.

† *P *<* *0.01 versus baseline.

**Table 4:** Summary of CO_2_ reactivity

	Study group		*P* value	
	Patients (*n* = 15 [*Af*] and 14 [*Un*])*	Controls (*n* = 14)	Group × P_ET_CO_2_	H × P_ET_CO_2_
Hypercapnia response	*Af* 2.1 ± 0.17 *Un* 2.1 ± 0.19	1.9 ± 0.20	0.64	0.96
Hypocapnia response	*Af* 2.9 ± 0.22 *Un* 2.8 ± 0.21	3.1 ± 0.23	0.53	0.51

Values are mean ± SE in % mmHg^−1^. Af, affected hemisphere; Un, unaffected hemisphere; P_ET_CO_2_, partial pressure of end-tidal CO_2_; H, hemisphere.

* For hypocapnia response. For hypercapnia, *n* = 14 affected hemisphere and 13 unaffected hemisphere of patients.

**Figure 3:**
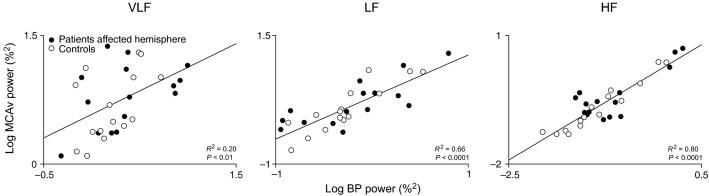
Bivariate linear correlations between BP and MCAv spectral powers. Group data are pooled (affected hemisphere in patients [*n* = 15] and controls [*n* = 15]). Results of multiple regression analysis with additional predictors are reported in text. *R*^*2*^ values are coefficients of determination. Data has been log-transformed and plotted on linear axes. BP, blood pressure; MCAv, middle cerebral artery flow velocity; VLF, very low-frequency; LF, low-frequency; HF, high-frequency.

To explore for possible determinants of CFV, we performed multiple regression analyses with MCAv power as a dependent variable, and BP power as an independent variable, with cohort status (i.e., patient or control) or PSI as additional predictors. Analysis of pooled data (controls and affected hemisphere of patients) indicated that TIA was not a significant determinant of CFV at all frequency ranges (all *P *≥* *0.63). However, in the LF range, PSI was predictive of CFV (*P *<* *0.05)(VLF and HF, *P *≥* *0.50). Finally, BP power was a positive predictor of MCAv power at all frequency ranges (VLF *R*^*2*^ = 0.20, LF *R*^*2*^ = 0.66, HF *R*^*2*^ = 0.80, all *P *<* *0.01). The corresponding bivariate linear correlations between BP and MCAv power are shown in Figure3.

## Discussion

This study assessed key markers of hemodynamic variability and CBF regulation in TIA. Contrary to our expectations, we found that cerebral perfusion stability is maintained in patients who have suffered a TIA due to intact cerebrovascular control. However, consistent with our hypothesis we found that BPV was higher in patients compared to controls. Since elevated BPV is an independent risk factor for stroke and poor neurological outcomes, our findings suggest that BPV is a potential therapeutic target for enhancing secondary stroke prevention in patients who suffer a TIA.

### Hemodynamic stability following TIA

Mounting evidence indicates that in addition to established risk factors like hypertension, having a *stable* hemodynamic profile is important for brain tissue integrity (Ko et al. 2010). Greater BPV in ischemic stroke is an independent risk factor for hemorrhagic transformation (Ko et al. 2010), and in patients with a previous TIA, elevated visit-to-visit systolic BPV is predictive of stroke (Rothwell et al. 2010). Consistent with this, impairment of CBF regulatory processes such as CA and CO_2_ reactivity are associated with worse outcomes (Grosset et al. 1993; Czosnyka et al. 1996; Budohoski et al. 2015). In this study, we found that patients who had suffered an episode of TIA had higher levels of BP spectral power across the 0.02–0.4 Hz range, indicating greater perfusion pressure instability compared to age-matched controls.

The mechanisms underpinning the elevation in BPV are likely to be complex and frequency dependent. Very low-frequency fluctuations are thought to originate from a combination of myogenic activity and humoral factors (renin–angiotensin system) (Stauss 2007). In contrast, low-frequency oscillations primarily reflect baroreflex and vascular sympathetic activity (Zhang et al. 2002; Stauss 2007), and high-frequency variability are related to mechanical effect of ventilation (Novak et al. 1993) and cardiac baroreflex function (Sin et al. 2010). Our observation that BPV was higher in each of these frequency bands are therefore consistent with previous studies showing clear associations between cardiovascular disease and autonomic dysfunction (Sykora et al. 2009), including baroreflex impairment (Creager and Creager 1994) and sympathetic hyperactivity (Grassi 2010). Another possibility is that BPV was increased secondary to the higher body mass index (Piccirillo et al. 1998). Moderate obesity, has been shown to increase HF systolic BPV, in the presence of normal average blood pressure (Piccirillo et al. 1998). We also noted that patients had higher mean systolic and diastolic blood pressure. Increases in average blood pressure correlate with greater BPV (Mancia et al. 1983), and may also explain the observed difference in BPV between patients and controls. Although we had hypothesized that BPV may be contributing to an increased risk of stroke by bringing perfusion close to thresholds for brain tissue integrity and function (Heiss and Graf 1994), our data suggest that CFV is not elevated, and that BPV is not a strong predictor of CFV. This does not imply that BPV is a not a risk factor, given there are alternative mechanisms by which BPV may cause vascular injury. Indeed, BPV may still contribute to mononuclear leukocyte endothelial adhesion and subsequent development of atherosclerosis (Chappell et al. 1998). Since high-risk cardiovascular cohorts commonly exhibit multiple comorbidities, the extent to which these blood pressure-related variables independently contribute to a patient’s overall risk warrants further investigation (Rothwell et al. 2010).

This study is the first to characterize CFV in TIA patients, but unlike BPV, we did not observe higher CFV. This seems to be consistent with our observation that vascular mechanisms that are normally involved in the dynamic stabilization of CBF appear to be intact in this patient cohort, in agreement with previous studies that did not find deficits in either CA (Atkins et al. 2010) or CO_2_ reactivity (Thompson 1971). Furthermore, in keeping with this inference we did not observe any differences in either CA or CO_2_ reactivity between the affected and unaffected cerebral hemispheres. However, we did observe a clear difference in HF transfer function phase between patients and controls. The precise mechanisms underpinning these differences are unclear, but it has been suggested that HF pressure-flow dynamics may reflect the impedance properties of the cerebrovasculature (Zhang et al. 1998; Zhu et al. 2011). If so, we speculate that the differences in HF phase may be due to differences in the viscoelastic properties of the cerebrovasculature associated with hypertension or chronic vascular disease.

### Hemodynamic determinants of CFV

To identify the key determinants of CFV, we conducted multiple regression analyses to quantify the extent to which BPV explained interindividual differences in CFV variance. Our analyses showed that the relations between BPV and CFV were clearly frequency dependent with only 20% of CFV variance explained in the VLF range, but up to 80% explained in the HF range. Furthermore, we considered the potential role of cohort status (i.e., presence or absence of prior TIA) and the integrity of CA as possible additional determinants, and found that TIA was not a significant predictor of CFV. In contrast, in the LF range, CA was a determinant of CFV, indicating our ability to account for CFV improves with integrated physiological measures of CBF control. In this regard, there is a documented association between cardiovascular disease and autonomic dysfunction (Sykora et al. 2009), and recent data suggest that cerebral sympathetic and cholinergic drive may be important determinants of CFV (Hamner et al. 2010, 2012; Peebles et al. 2012). However, defining the precise role of cerebral autonomic modulation in patient cohorts would be challenging since cerebral autonomic activity can only be assessed using highly invasive methodologies such as transcranial plasma norepinephrine spillover (Mitchell et al. 2009) or autonomic blocking studies (Ogoh et al. 2010).

### Methodological considerations

The results of this study should be interpreted in cognisance of several methodological considerations. First, we employed wavelet phase synchronization analysis to assess CA as it offers several theoretical advantages. Conventional metrics such as transfer function analysis are inherently linear, and assume that pressure-flow dynamics are stationary over time; an assumption that in practice, is likely to be violated (Latka et al. 2005). Moreover, these approaches typically do not account for the influence of arterial PCO_2_ on the pressure-flow relationship (Peng et al. 2010), which may be considerable at low frequencies (Mitsis et al. 2004). Such shortcomings may underlie why convergent validity is poor between traditional metrics, and dissimilar conclusions may be drawn in response to common physiological perturbations (Tzeng et al. 2012). For these reasons, we adopted a nonlinear and nonstationary approach based on wavelet analysis that also accounts for the confounding effects of arterial PCO_2_ on pressure-flow dynamics.

Second, we assessed patients at approximately between 1 and 2 weeks after their initial presentation. This was done to ensure that our hemodynamic assessments were undertaken after the initiation and stabilization of current secondary prevention therapies.

Finally, transcranial Doppler measures cerebral blood flow velocity, which is only a valid surrogate of volumetric flow providing the cross-sectional area of the insonated vessel remains constant (Serrador et al. 2000). In this study, we did not obtain direct MRI measurements of MCA diameter to verify this assumption. But, recent high-field MRI studies indicate that the MCA diameter versus P_ET_CO_2_ relationship is magnitude dependent. MCA diameter changes at +9 and −13 mmHg P_ET_CO_2_ (Coverdale et al. 2014), but not within the ±7.5 mmHg range (Verbree et al. 2014). Based on this evidence, we perturbed P_ET_CO_2_ to ∼5 mmHg above and below resting baseline levels to ensure that our Doppler assumptions can be reasonably justified against current evidence.

### Implications

TIA is a harbinger of stroke and therefore episodes of TIA offer a crucial opportunity to intervene. Greater BPV is a potential risk factor for poor outcomes (Parati et al. 2013), but its relevance in TIA is poorly understood. Here, we have shown that TIA patients have higher BPV approximately 1–2 weeks after presentation, suggesting this risk factor remains a potential therapeutic target. However, the effects of antihypertensive therapy on BPV remains poorly understood and current blood pressure treatments protocols are primarily focused on reducing absolute blood pressure. One potential agent that should be considered is calcium channel blockers, which have been shown to selectively reduce BPV when administered in relatively low doses (Zhang et al. 2011; Tzeng and Macrae 2013). Since technologies to noninvasively monitor BPV (e.g., finger plethysmography, ambulatory blood pressure monitors) already exist, future studies are needed to assess if this higher BPV can be effectively targeted to reduce the incidence of poor neurological outcomes following TIA.

## Conclusion

Our study shows that patients who have suffered a TIA have greater BPV. We identified BPV as a key determinant of CFV and suggest that hemodynamic instability may contribute to the elevation of stroke risk in this clinical cohort. Longitudinal studies are needed to establish whether the assessment and attenuation of hemodynamic variability in patients presenting with TIA may be beneficial in preventing progression to stroke.

## Appendix

### Wavelet Phase Synchronization Analysis

The complex wavelet transform of a signal *s*(*t*) can be found by:

1

with a complex mother wavelet *ψ*(*t*). *a* is the scale creating translation and dilation of the mother wavelet, and *t*_0_ is the time-index sweeping through the time-series. The instantaneous phase of the signal *s*(*t*) can be readily extracted from its wavelet transform *W*_s_(*a*, *t*_0_) using:

2

Herein, we employ complex Mortlet wavelet:

3

with the centre frequency *f*_c_, and the bandwidth parameter *f*_b_*,* both set to 1. The scale *a* is associated with a pseudo-frequency by:

4

with the sampling period *δt*, also equal to 1. For single-input single-output model, the phase difference between BP-MCAv is:

5

where *Φ*_P_ and *Φ*_V_ are wavelet-based instantaneous phases of BP and MCAv, respectively. The synchronization index is calculated by:

6

where *N* is the number of data points in each time series, and Δ*Φ*_PV_(*a*, *t*_0_) is the instantaneous phase difference between BP and MCAv time series, given in equation 5.

For two-input single-output (BP-MCAv with CO_2_) model, the phase difference will be:

7

where *Φ*_PC_ is wavelet-based phase difference of BP-CO_2_, and *Ψ*_CV_ and *Ψ*_PV_ are TFA-based phase differences of CO_2_-MCAv and BP-MCAv, respectively i.e., ; *H*_CV_ and *H*_PV_ are the transfer functions of CO_2_-MCAv and BP-MCAv, respectively. *W*_C_ and *W*_P_ are the wavelet-coefficients using equation 1 for CO_2_ and BP, respectively. In equation 7 the first term [i.e., Δ*Φ*_PV_(*a*, *t*_0_)] represents the wavelet-based phase difference between BP-MCAv, and the second term is the identified phase error caused by CO_2_. Δ*Φ*_PV_(*a*, *t*_0_) shows the corrected phase between BP-MCAv after incorporating the effects of CO_2_ in equation 7. The synchronization index for corrected phase difference (eq. 7) is:

8

### Transfer Function Analysis

In this study decimated (1 Hz) waveforms were passed through a Hanning window before undergoing linear transfer function analysis using the Welch technique (50% overlap). The transfer function H (*f*) between BP and MCAv signals was calculated as:

H (*f*) = *S*_xy_ (*f*)/*S*_xx_(*f*)

where *S*_xx_ (*f*) is the autospectrum of input signal (BP) and *S*_xy_ (*f*) is the cross-spectrum between the two signals (BP and MCAv). Transfer function magnitude ∣H(*f*)∣ and phase spectrums ∣*Ф* (*f*)∣ were obtained from the real and imaginary parts of the complex transfer function:

∣H(*f*)∣ = {[*H*_R_ (*f*)]^2^ + [*H*_I_ (*f*)]^2^}

Ф (*f*) = tan^−1^[*H*_I_ (*f*)/*H*_R_ (*f*)]

The squared coherence function MSC (*f*) was defined as:

MSC (*f*) = ∣*S*_xy_ (*f*)∣^2^/[*S*_xx_(*f*)*S*_yy_(*f*)],

where *S*_yy_(*f*) is the autospectrum of changes in output signal (MCAv).

The relative amplitude and time relationship between BP and MCAv_mean_ are reflected as transfer gain and phase shifts over a specific frequency range. To establish summary statistics, the coherence, gain and phase measures were band averaged within the VLF, LF and HF ranges.
